# Evaluation of Marginal/Internal Fit and Fracture Load of Monolithic Zirconia and Zirconia Lithium Silicate (ZLS) CAD/CAM Crown Systems

**DOI:** 10.3390/ma14216346

**Published:** 2021-10-23

**Authors:** Haneen A. Sadeqi, Mirza Rustum Baig, Mohammad Al-Shammari

**Affiliations:** 1 Department of Bioclinical Sciences, Faculty of Dentistry, Kuwait University, P.O. Box 24923, Safat 13110, Kuwait; haneen.sadeqi@ku.edu.kw (H.A.S.); mohammad.alshammari@ku.edu.kw (M.A.-S.); 2 Department of Restorative Sciences (Prosthodontics), Faculty of Dentistry, Kuwait University, P.O. Box 24923, Safat 13110, Kuwait

**Keywords:** monolithic, zirconia, lithium silicate, ceramic, fracture load, marginal fit, internal gap, CAD/CAM, dynamic loading, nano-CT evaluation

## Abstract

Fit accuracy and fracture strength of milled monolithic zirconia (Zi) and zirconia-reinforced lithium silicate (ZLS) crowns are important parameters determining the success of these restorations. This study aimed to evaluate and compare the marginal and internal fit of monolithic Zi and ZLS crowns, along with the fracture load, with and without mechanical aging. Thirty-two stone dies acquired from a customized master metal molar die were scanned, and ceramic crowns (16 Zi Ceramill Zolid HT^+^ and 16 ZLS Vita Suprinity) were designed and milled. Absolute marginal discrepancies (AMD), marginal gaps (MG), and internal gaps (IG) of the crowns, in relation to the master metal die, were evaluated using x-ray nanotomography (*n* = 16). Next, thirty-two metal dies were fabricated based on the master metal die, and crowns (16 Zi; 16 ZLS) cemented and divided into four groups of eight each; eight Zi with mechanical aging (MA), eight Zi without mechanical aging (WMA), eight ZLS (MA), and eight ZLS (WMA). Two groups of crowns (Zi-MA; ZLS-MA) were subjected to 500,000 mechanical cycles (200 ± 50 N, 10 Hz) followed by axial compressive strength testing of all crowns, until failure, and the values were recorded. Independent sample *t* tests (α = 0.05) revealed no significant differences between Zi and ZLS crowns (*p* > 0.05); for both internal and marginal gaps, however, there were significant differences in AMD (*p* < 0.005). Independent samples Mann–Whitney U and Kruskal–Wallis tests revealed significant differences between the two materials, Zi and ZLS, regardless of fatigue loading, and for the individual material groups based on aging (α = 0.05). Multiple comparisons using Bonferroni post-hoc analysis showed significant differences between Zi and ZLS material groups, with or without aging. Within the limitations of this study, the ZLS crown fit was found to be on par with Zi, except for the AMD parameter. As regards fracture resistance, both materials survived the normal range of masticatory forces, but the Zi crowns demonstrated greater resistance to fracture. The monolithic Zi and ZLS crowns seem suitable for clinical application, based on the fit and fracture strength values obtained.

## 1. Introduction

Complete coverage ceramic crowns are being routinely used in dentistry as a substitute for the traditional ceramo-metal options for multiple reasons, including fine aesthetics [1,2]. Different types of zirconia-based ceramics have been made available in recent years for the restoration and replacement of both anterior and posterior teeth through a variety of fabrication techniques, including computer-aided design and computer-aided manufacture (CAD/CAM) [3,4]. Although zirconia (Zi) has favorable mechanical properties for a variety of applications in dentistry, the white color and poor translucency of the material precluded the use for full contoured restorations in the past [5,6,7,8,9]. Recently, translucent tooth-colored Zi, which enables fabrication of restorations without the veneering porcelain, has been developed [10,11,12]. Lithium disilicates have also been in wide use for making complete coverage crowns in dentistry [13,14], and the material can be either pressed or milled by CAD/CAM. Recently, a modified form of this material, zirconia-reinforced lithium silicate (ZLS) was introduced [6,15]. The ZLS material has the lithium silicate crystals in a glassy matrix in combination with 8–12% zirconia crystals, which act to inhibit crack propagation and increase fracture resistance through phase transformation [16]. Fracture strength and fatigue behavior of ceramics under intra-oral occlusal loading are crucial pre-determinants of restoration success. Multiple factors related to the restoration, supporting structure (substrate), cementation, and oral environment have been identified in literature as influencing the crown fracture load [1,2,5,8,16]. Some specific restoration-based factors are the ceramic material composition and properties, internal fit, processing variations, crown dimensions/geometry, and finishing/glazing effect. The substrate-related reasons include modulus of elasticity and preparation design [10,11,12,16]. Although many papers have examined the fracture load of different types of ceramic restorations, data are scarce on the in-vitro fatigue performance and fracture load of single posterior crowns made with the new monolithic translucent Zi and ZLS ceramic materials.

Marginal fidelity is a key parameter used to gauge clinical acceptability of fixed restorations, and also of clinical success at post-placement evaluations [1,9,17,18]. McLean et al. [19] suggested 120 µm as a clinically acceptable marginal discrepancy for ceramic crowns, and the value fits the overall range of 4–174 µm reported by systematic reviews [20,21] for ceramic crowns. Investigators have reported that sizeable marginal gaps can possibly cause complications such as periodontal inflammation, cement breakdown, recurrent caries, and even irreversible pulpal damage [22,23,24]. Several factors have been implicated as potentially affecting the fit accuracy of ceramic crowns, including the type of CAD/CAM and milling system, measurement method used, number of measure sites, ceramic material type, and preparation design used, amongst other variables [20,21]. A number of studies have assessed the fit accuracy of CAD/CAM ceramic crowns and found varied outcomes [20,21,25,26,27]. Still, research on the marginal and internal adaptation of monolithic zirconia CAD/CAM crowns has mostly been restricted and is especially lacking with the anatomically contoured translucent partially stabilized zirconia (PSZ) and ZLS crowns [20,21,28,29]. To the authors’ best knowledge, studies evaluating the fit accuracy of complete coverage crowns using nano-computed tomography (CT) methods are also rare, although micro-CT evaluation of indirect fixed restorations has been reported in several papers [28,30,31].

This in vitro study aimed to evaluate the accuracy of fit of CAD/CAM complete coverage monolithic ZLS crowns and compare with monolithic Zi crowns, in terms of marginal gap (MG), absolute marginal discrepancies (AMD), and internal gap (IG) widths between the crowns and the master die (conforming to a mandibular molar tooth preparation), by using nano CT-based analysis. Secondly, the study aimed to assess the fracture resistance of Zi crowns compared to the ZLS crowns, with and without mechanical aging (cyclic loading). The null hypotheses tested were that there would be no differences in the accuracy of fit, in terms of marginal and internal gap, or with respect to the fracture resistance, between monolithic Zi and ZLS crowns. The second null hypothesis was that there would be no difference between the mechanically aged and non-aged crowns, in terms of fracture loads, for both Zi and ZLS materials.

## 2. Materials and Methods

### 2.1. Preparation of the Master and Working Dies

**Figure 1:**
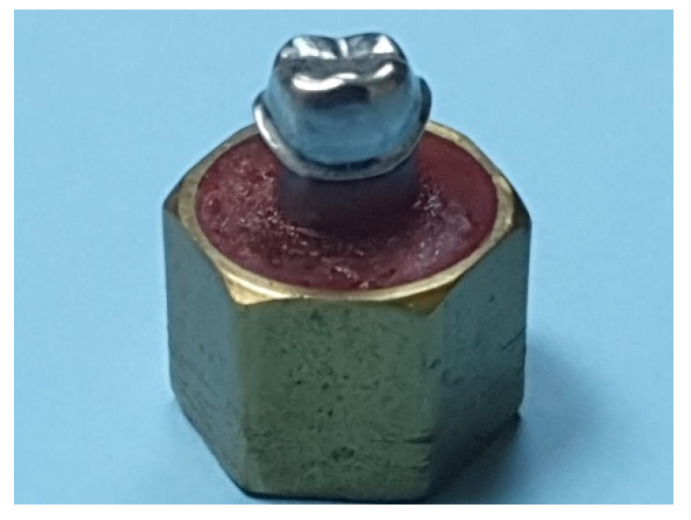
Master metal die.

An ivorine mandibular first molar tooth (Columbia Dentoform Corp, Long Island City, NY, USA) was prepared using a high-speed handpiece (KaVo Bella Torque Mini; KaVo, Lake Zurich, IL, USA) and a 3-degree diamond rotary cutting bur (847 KR 016 NTI Diamond Instruments; Kahla–GmbH, Thuringia, Germany) to achieve a 1-mm wide continuous rounded shoulder. An occlusal reduction of 2 mm and 1.5 mm were achieved on the buccal and lingual cusps and a cervico-occlusal wall height of at least 4 mm was maintained on all the axial surfaces of the abutment. The overall occlusal convergence of the preparation was kept below 20 degrees. The prepared ivorine tooth was duplicated and cast using cobalt-chrome (Co-Cr) alloy (Solidur CoCr, dental alloy, YETI Dentalprodukte GmbH, Engen, Germany). The resultant metal master die was polished, finished (Silicone carbide stones-Dura greens and Silicone Hardies, Shofu finishing and polishing systems; Shofu Inc, Kyoto, Japan), and mounted into a machined brass cylinder and held in position with the aid of self-cure acrylic resin (GC Pattern resin; GC Corp, Tokyo, Japan) (Figure 1). Thirty-two impressions of the Co-Cr alloy master die were made using vinyl polysiloxane (light body and regular body; 3M ESPE, Express XT, Neuss, Germany) impression material in custom trays (Preci Tray; YETI Dentalprodukte GmbH, Engen, Germany) and poured using type V dental stone (Jade stone; WhipMix, Louisville, KY, USA). The dies were retrieved from the impressions after 30 min and allowed to set for a further 24 h. All the stone dies were checked visually and examined under the microscope (10X, Leica microsystems, model A60, Singapore, Singapore), and if found defective with voids or other surface irregularities, were discarded and redone using new impressions. The 32 stone dies were randomly (by numbering and drawing lots) allocated to two groups of 16 dies each, where each group was specifically assigned to a crown system Zi (Ceramill Zolid HT^+^, Amann Girrbach, Pforzheim, Germany) and ZLS (Vita Suprinity HT, Ivoclar Vivadent AG, Bad Säckingen, Germany). This resulted in two groups of 16 Zi and 16 ZLS die specimens [25,32,33,34,35]. Additionally, 32 universal (Co-Cr) metal dies were also prepared from the master metal die. The 32 metal dies were modeled on the master metal die using the duplication process and cast in Co-Cr alloy (Solidur CoCr, YETI Dentalprodukte GmbH, Engen, Germany).

### 2.2. Preparation of Test Samples

A complete contour wax-up was completed on one of the stone die samples for use as a template for the fabrication of standardized anatomically contoured monolithic crowns (Zi and ZLS). The stone die was scanned with the wax-up using a laboratory scanner (Ceramill MAP400, Amann Girrbach, Koblach, Vorarlberg, Austria), and 3D scan image created. Next, the two groups of stone dies were scanned using the same laboratory scanner (Ceramill MAP400, Amann Girrbach) to generate 32 individual scans (n = 16). The dies were sprayed with occlusal spray (YETI Dentalprodukte, GmbH, Engen, Germany) prior to scanning and a silicone platform was created to place the dies in the scanner in the same position for all scans. The wax-up 3D scan image was then superimposed on the individual stone die scans using ‘scan pre-op model’ option to design identical uniformly contoured virtual complete coverage crowns using CAD software (Ceramill Mind 3.4.7, Amann Girrbach), with a 0.05 mm cement space setting. The minimum thickness of the crown was set at 1 mm. The 3D data were then used to instruct a 5-axis milling machine (Ceramill motion 2, Amann Girrbach) to mill a total of 16 Zi crowns by dry milling and 16 ZLS crowns by wet milling, respectively, using Zi (Ceramill Zolid HT^+^ White, Amann Girrbach) and ZLS (Suprinity HT, Vita Zahnfabrik, Bad Säckingen, Baden Württemberg, Germany) blocks and specific types of milling bits and grinding pins for each material according to the manufacturer’s recommendation. (Table 1). The manufactured crowns were cleaned with an extra-fine brush to remove any residual powder that might have settled on the intaglio or occlusal surfaces of the crowns from the milling process. All fabricated crowns were checked carefully under the microscope (10X, Leica microsystems, A60, Singapore, Singapore) for any defects, cracks, or chipped areas that may have resulted from milling. The Zi monolithic crowns were then placed in a sintering furnace (Ceramill therm, Amann Girrbach, Koblach, Vorarlberg, Austria) at a maximum temperature of 1450 °C for 8 h, with a firing paste, for the sintering procedure. The ZLS crowns were fired in the ceramic porcelain furnace (P310, Ivoclar vivadent AG, Schaan, Leichtenstein) at a final temperature reaching 840 °C for about 15 min to achieve full crystallization. Crown margins were then assessed again under the microscope (10X, Leica microsystems, A60, Singapore). Both types of crown specimens were finished, polished (Polishing set, Vita Zahnfabrik GmbH, Bad Säckingen, Germany), and glazed, at 850 °C for 6 min for Zi crowns (P310, Ivoclar vivadent AG, Schaan, Leichtenstein), and 800 °C for 10 min (P310, Ivoclar vivadent AG, Schaan, Leichtenstein) for ZLS crowns. A total of 32 crowns were thus fabricated for marginal and internal fit evaluation: Group 1, 16 monolithic Zi crowns; Group 2, 16 monolithic ZLS crowns (n = 16). The sample size of this study was based on earlier related published studies [25,28,32,33,34,35,36,37,38]. Based on the mean differences and standard deviation assumptions, the total sample size was estimated as 24 crown samples (12 for each of the two ‘ceramic material’ groups), at α = 0.05 and power of 85% (G* Power statistical power software v.3.1.9.7) for the marginal and internal gap evaluations. For the fracture load, the sample size was again calculated at 8 samples per test group to detect differences, based on earlier studies [28,29] to achieve a power of 85% at α = 0.05. So, a total of 16 crowns were evaluated for each material group and 8 crowns for each aging-based sub-group in this study.

### 2.3. Evaluation of Marginal/Internal Fit by Nano-CT

**Figure 2:**
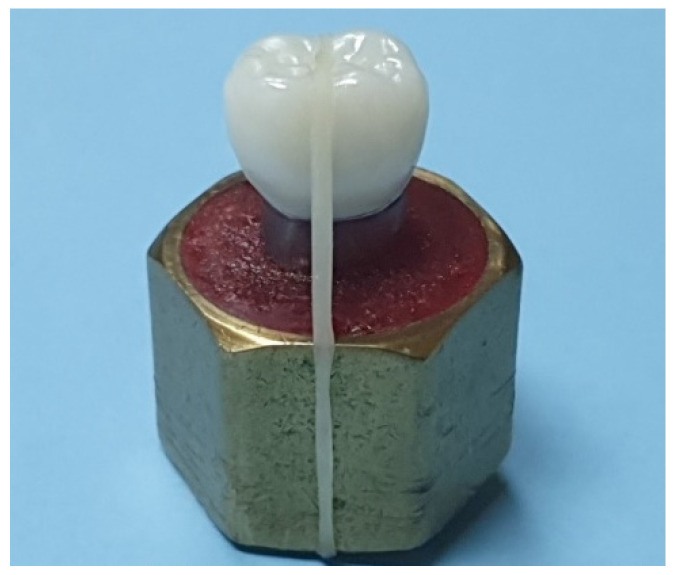
Elastic band holding the crown on the master metal die.

All the crowns belonging to both the groups were individually seated on the master metal die and secured with an orthodontic intraoral elastic band (diameter 6.3 mm, heavy stark 170 g = 6 OZ, Forestadent, Pforzheim, Germany) prior to fit evaluation, using X-ray nanotomography (Phoenix Nanotom-M3D nanoCT; GE GmbH., Solingen, Germany). The elastic band passed over the occlusal surface of the crown and the abutment cylinder base and aided in preventing any displacement of the crown from the master metal die and in maintaining adequate pressure on the crown during the fit evaluation procedure (Figure 2). For each crown, a new elastic band was used.

The abutment-crown assembly was carefully positioned perpendicular to the X-ray source using a platform that was used for all the samples to standardize the scan position. The parameters of the nano-CT machine were setup as illustrated in Table 1. Two thousand images were obtained for each scan per crown-abutment sample and reconstructed using CT software (Phoenix, datoslx v. 2.3.3; GE GmbH.,Solingen, Germany). The initial reconstructed image was assessed and upon approval, a final X-ray 3D model of the crown-abutment sample was generated using data view software (VGStudio Max v 3.1, Volum graphics, Heidelberg, Germany), and the sagittal slices were isolated from the reconstructed images. Before performing the definitive measurements on the test samples, the measuring device (nano-CT equipment with software) was calibrated for precision and accuracy by analyzing the system error. A single abutment-crown sample was scanned five times consecutively without removing it from the target platform and by retaining in the same position in the nano-CT machine. Marginal gap measurements were performed on corresponding sagittal sections of all the five virtual models to determine the differences between the repeated scans at set locations. The discrepancies found were below 5 microns between the different 3D models. The trueness of the device was then assessed using a ball-bar CT scan artifact, in relation to the values obtained using a scientific digital caliper and co-ordinate measuring machine (CMM) multiple times. The deviations were below 10 microns. The accuracy of the nano-CT system was found to be within acceptable limits for conducting the fit evaluation exercise.

**Figure 3:**
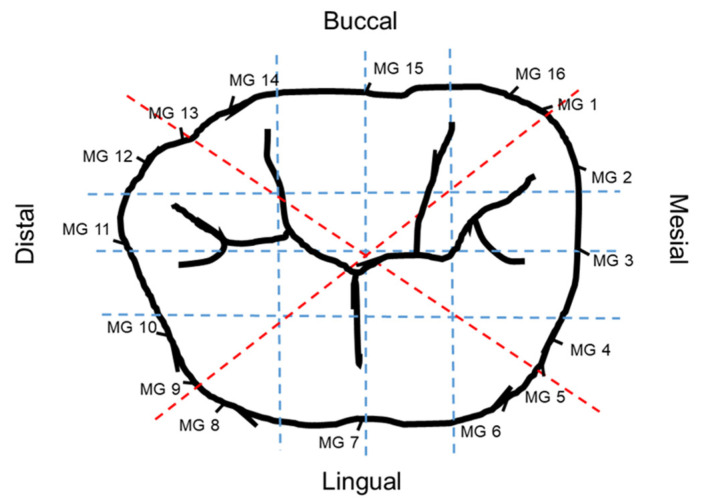
Schematic diagram (occlusal view of the crown) showing the different mesio-distal and bucco-lingual sagittal sections used for crown marginal and internal gap evaluation, represented by blue and red dotted lines. MG 1 to MG 16 represent the marginal gap measurement sites all around the crown for different sections.

Three different sagittal and coronal slices were selected both bucco-lingually (BL) and mesio-distally (MD), in addition to two slices that were chosen from the mesio-buccal (MB) to disto-lingual (DL) corner and disto-buccal (DB) to mesio-lingual (ML) corner, as shown in Figure 3.

**Figure 4:**
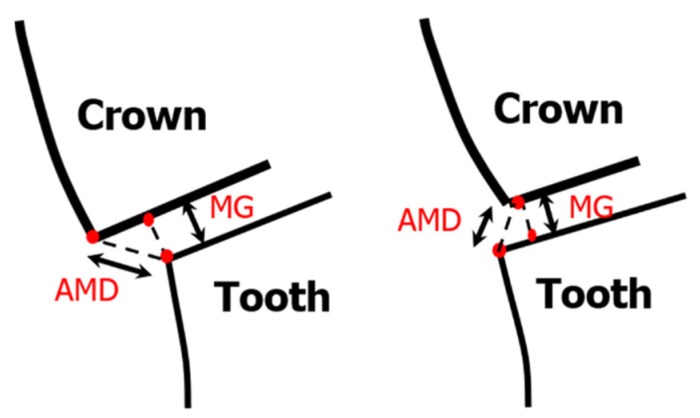
Schematic diagram showing MG and AMD measurement scheme at the crown-abutment junction for positive and negative overhang.

**Figure 5:**
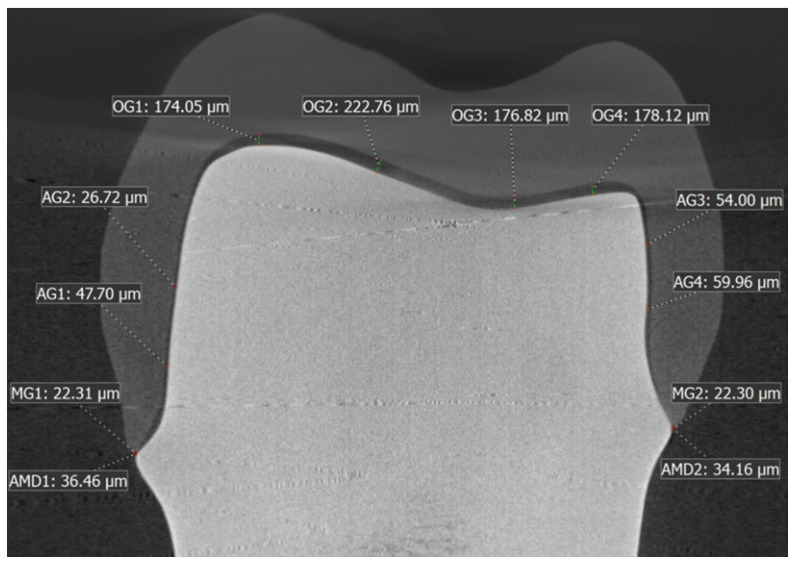
Representative image of the crown-master metal die bucco-lingual section of the nano-CT 3D scan model showing the internal and marginal gap width measurements.

The slices were set at equidistant intervals (distances) of 2700 µm, 0.00 µm, and −2700 µm on the 3D model respectively for standardization purposes for all crowns, with 0.00 µm position being the center of the crown. The greatest mesio-distal and bucco-lingual widths of the abutment at the finish line level were 11 mm and 9 mm, respectively. In each of the six bucco-lingual and mesio-distal slices, 10 locations were chosen for measurement of marginal and internal fit [26,28,39]. AMD and MG were evaluated at the crown-abutment margin locations based on the method suggested by Holmes et al. and applied by several other studies [28,31,34] (Figure 4). Gaps were recorded at four locations on the axial wall, four locations on the occlusal wall, and two locations on the margins denoting AMD and MG, as shown in Figure 5.

The locations for internal gap measurement were chosen to be at equidistant intervals of 1.5 mm distance starting from the finish line margin on the axial wall, and from the mesial occluso-axial line angle on the occlusal wall, for any given slice. In the last two slices connecting the different axial line angles, only two locations were chosen at the margins for evaluation of AMD and MG. Hence, for each crown sample, there were 16 AMD and MG values recorded along with 48 internal gap widths in the occlusal and axial regions. All measurements pertaining to the marginal and internal fit of the Zi and ZLS crowns were recorded in µm. All measurements were performed by the same investigator for each of the 8 sagittal slices at 16 marginal locations and 48 internal gap locations. For the AMD and MG, three measurement repetitions were performed at each of the marginal evaluation sites and the average value was used. One pilot sample each of the ZLS and Zi crowns was randomly selected, and AMD and MG values were recorded three times at each marginal location site for a given sagittal slice, prior to the actual marginal fit evaluation to assess intra-operator reliability. The results showed a high intraclass correlation co-efficient of >0.93 for AMD and MG.

### 2.4. Cementation of Crown Samples on Metal Dies

The 32 crowns that were previously used for marginal and internal fit evaluation were fitted individually on the universal metal die replicas and checked for marginal accuracy and internal adaptation, using visual and tactile examination with dental explorer by applying modified United States Public Health Services (USPHS) and California Dental Association (CDA) criteria for marginal fit assessment of crowns and fixed partial dentures (FPDs). The marginal fit was considered acceptable when there was no visible or presence of a slightly soundable gap, with no penetration of explorer (USPHS), and with or without crevice and catch along margin, but no penetration by explorer (CDA). The internal fit was deemed adequate when the crown was stable on the metal die without any rocking in mesio-distal or bucco-lingual directions. Additionally, silicone fit checker was used on the intaglio surface to further confirm the internal fit. If the crown was found to be of satisfactory fit, it was selected for cementation on individual dies [40,41,42,43,44]. Thirty-one crowns satisfied the criteria for marginal and internal fit, whereas one Zi crown was newly fabricated, as it did not meet the required criteria. All the crowns were steam cleaned (Steam generator, Steamer X3 Amann Girrbach, Koblach, Austria) and placed in an ultrasonic bath for 1 min (Pro-Sonic, Sultan, York, PA, USA), then removed and dried prior to the cementation [37]. The crowns were luted on the metal dies [45,46,47] using self-adhesive resin cement (RelyXTM U200, 3M ESPE, St. Paul, MN, USA), all by the same operator, and finger pressure maintained for 2 min, followed by a 2.2 kg static pressure load, which was maintained for 5 min [48]. Excess cement was removed using hand instruments from the crown-abutment junction. Photopolymerization was completed with Ivoclar light curing machine at 20 s per surface (mesial, distal, lingual, and buccal) using an LED source with 600 mW/cm^2^ at 10 mm distance (Targis Power, Ivoclar vivadent AG, Schaan, Liechtenstein). Subsequently, the metal dies were embedded in poly methyl methacrylate resin (PMMA) (Orthoplast acrylic resin, Vertex, Soesterberg, Netherlands), contained in a cylindrical brass mold, and vertically positioned until PMMA resin was set for easy handling and retention of the crown-abutment complex when subjected to fatigue and load-to-failure tests. Prior to the actual testing, all 32 samples (16 Zi and 16 ZLS crowns) cemented on abutment metal dies were stored by immersing in distilled water at room temperature for at least 1 week [32,48,49].

### 2.5. Fatigue Testing (Cyclic Loading)

**Figure 6:**
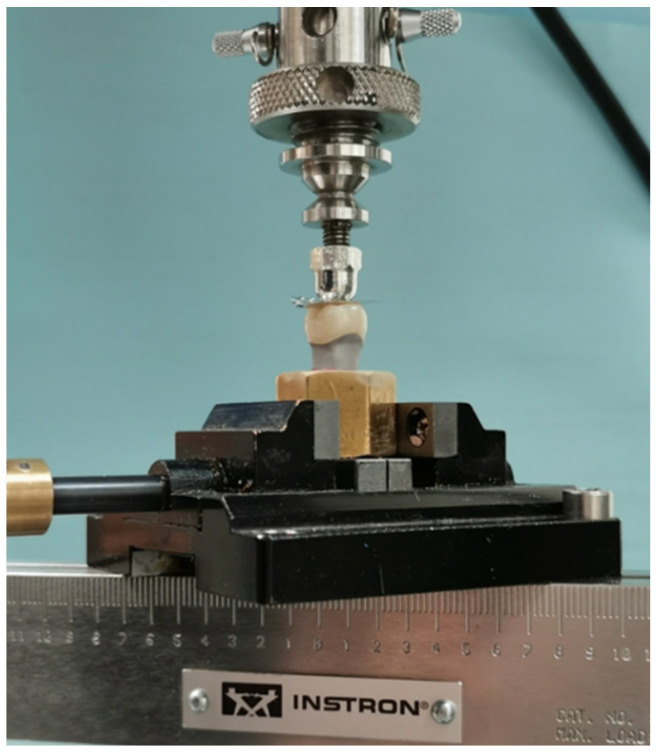
Crown set up for cyclic loading in the electro-dynamic testing system.

In each of the crown material groups (n = 16), the specimens were sub-divided into eight samples each. One sub-group underwent mechanical aging (MA) by compressive cyclic loading [37,48,50,51], and the other sub-group, which served as the control, did not undergo any aging process. The compressive cyclic loading of crowns (with occlusal thickness of 1.5–2 mm and rounded occlusal notch design) was performed using an electro-dynamic universal testing machine (UTM) (Instron ElectroPuls E3000, Instron Corporation, Norwood, MA, USA) in unidirectional movements along the long axis of the crown-abutment complex with a 5.0 mm diameter hardened hemi-spherical steel ball indenter centered at the central fossa of the occlusal surface to have a two-point contact, at a cusp angle of 70° [28] (Figure 6). A customized specimen holder device was used to ensure all specimens were placed in the same position for testing. Cemented crown specimens along with their bases were held in position in the loading device and subjected to 500,000 load cycles of 200 ± 50 N at 10 Hz [37,46,48,50,51,52,53]. The number of cycles used simulated a 2 year functional loading period in the mouth at an estimated rate of 240,000 cycles/year [54,55]. The Instron machine was programmed to stop if force dropped by 40%. After completion of the test cycle, each crown was checked under the microscope (10X, Leica microsystems, A60, Singapore, Singapore) for surface cracks and other irregularities.

### 2.6. Load to Fracture

Following the mechanical aging of eight crowns from each group, all 32 specimens were tested for load to fracture using a universal testing machine (Instron ElectroPuls E3000 & 5581, Instron Corporation, Norwood, MA, USA) until complete fracture. The compressive force was selected to be at a crosshead speed of 0.5 mm/min until failure. The semi-spherical steel head indenter of 5 mm diameter was placed in the central fossa of the occlusal surface. A thick (0.1 mm) piece of tin foil (Dentaurum GmbH, Ispringen, Germany) [47,56,57] was placed between the loading piston and the occlusal part of the crown to prevent loading stress peaks on the ceramic material surface. Fracture was defined as the appearance of visible cracks along with load drops (set at 40% drop in the maximum loading force) in the stress-strain diagram and acoustic occurrences. The fracture load value was recorded with the relevant software, and the first drop was marked as corresponding load at failure. The maximum load necessary to fracture each specimen was recorded in Newtons (N) [42,44,58]. The mode of failure of crowns was recorded according to a classification method [58,59] as follows:

Type I: minimal fracture or crack in the crown.
Type II: Loss of less than half of the crown.
Type III: Crown fracture through midline with half the crown lost.
Type IV: Severe fracture of the crown.

### 2.7. Stereomicroscopic Analysis of the Fractured Samples

A few samples were randomly selected to analyze the pattern of crack formation in more detail, under the stereomicroscope (5-10X, Stereo Discovery V12, Carl Zeiss, Jena, Germany).

### 2.8. Statistical Analysis

Data were analyzed using statistical software (SPSS 25, SPSS Inc., Chicago, IL, USA) and checked by an independent statistician for accuracy of performed tests and interpretation of results. Mean and SD values of AMD, MG, and IG were calculated. Normal distribution and homogeneity of data for AMD, MG, and IG were assessed using Kolmogorov–Smirnov and Shapiro–Wilk tests and verified. Levene’s test for equality of variances and independent student t-tests were used to analyze the data for AMD, MG, and IG (α = 0.05). For the fracture load values, normality of data was checked again for both material groups, and for the four material sub-groups (based on aging of crown samples). Based on the results, Mann–Whitney U-test and Kruskal–Wallis non-parametric tests were employed to test the effect of ‘material’ and ‘aging’ on the fracture load. The data were further analyzed using Bonferroni multiple comparison post-hoc tests to test the individual differences between and within material groups (α = 0.05).

## 3. Results

**Figure 7:**
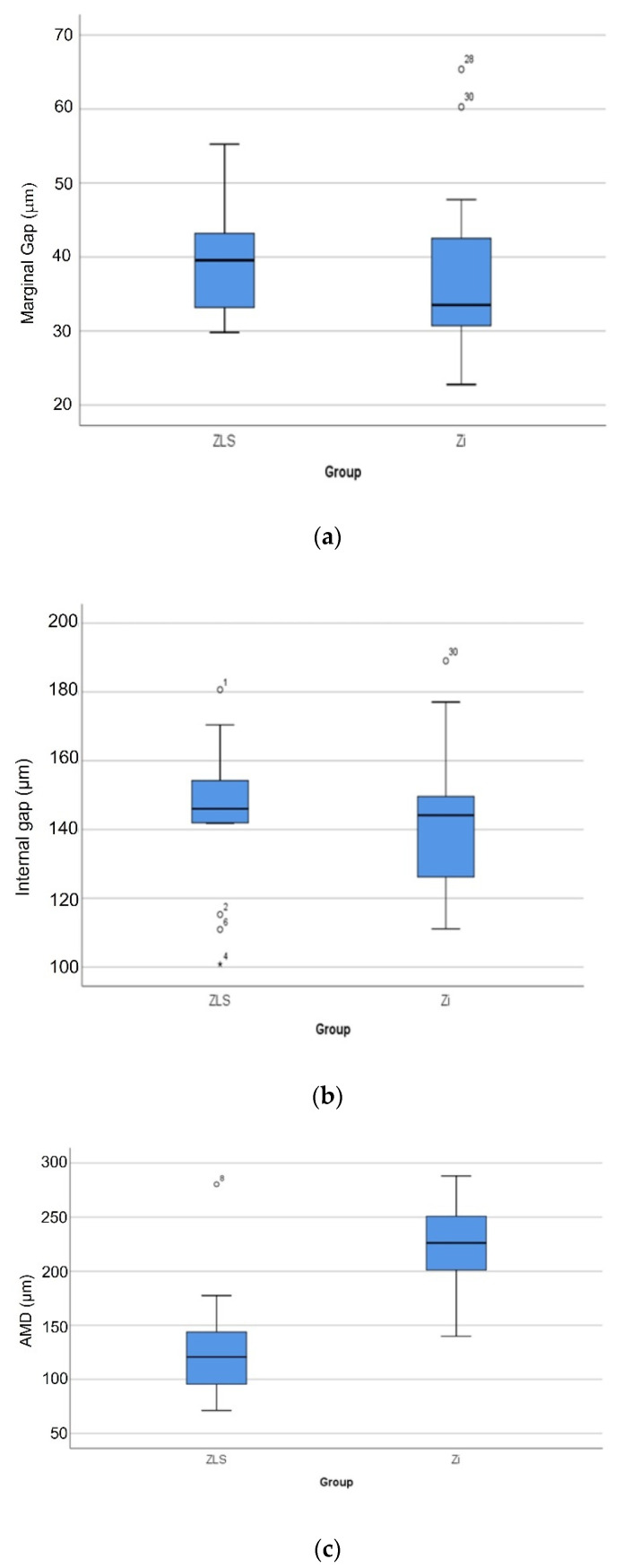
Box plot graphs showing the distribution of marginal gap (**a**), internal gap (**b**), and AMD (**c**) values. Circles and stars denote outliers with the test sample numbers displayed.

Table 2 lists the overall Mean ± SD values of MG, IG, and AMD for all the marginal and internal fit measurement locations combined, for Zi and ZLS crowns. The box plots (Figure 7a–c) show the distribution of the marginal gap, internal gap, and AMD data for the two material groups, through five statistics: minimum, first quartile, median, third quartile, and maximum. Using Student’s t-test, the differences in mean marginal gap and internal gap widths between Zi and ZLS crowns were not found to be significant (*p* > 0.05) (Table 3). However, significant differences were found between the mean AMD values of Zi and ZLS crowns (*p* < 0.05) (Table 3). With regard to the internal gap widths, the mean axial gap (AG) values were markedly lower than the mean occlusal gap (OG) values for both Zi and ZLS crowns. The numerical differences between the two materials for the two internal gap widths were, however, small (Zi AG—68.38 µm; ZLS AG—66.08 µm; Zi OG—214.84 µm; ZLS OG—225.58 µm).

**Figure 8:**
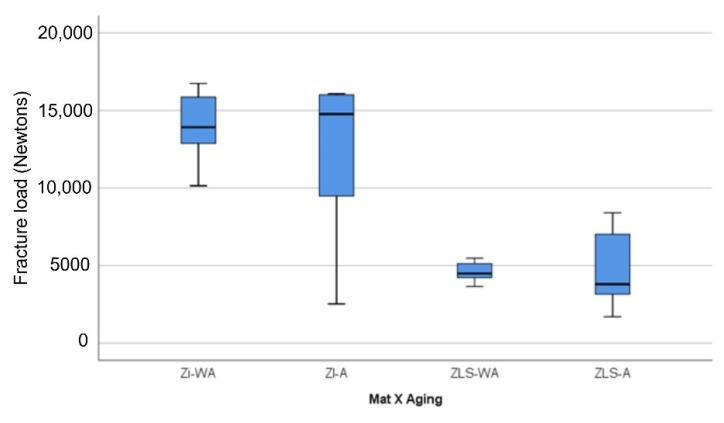
Fracture loads for the different material—aging groups.

**Figure 9:**
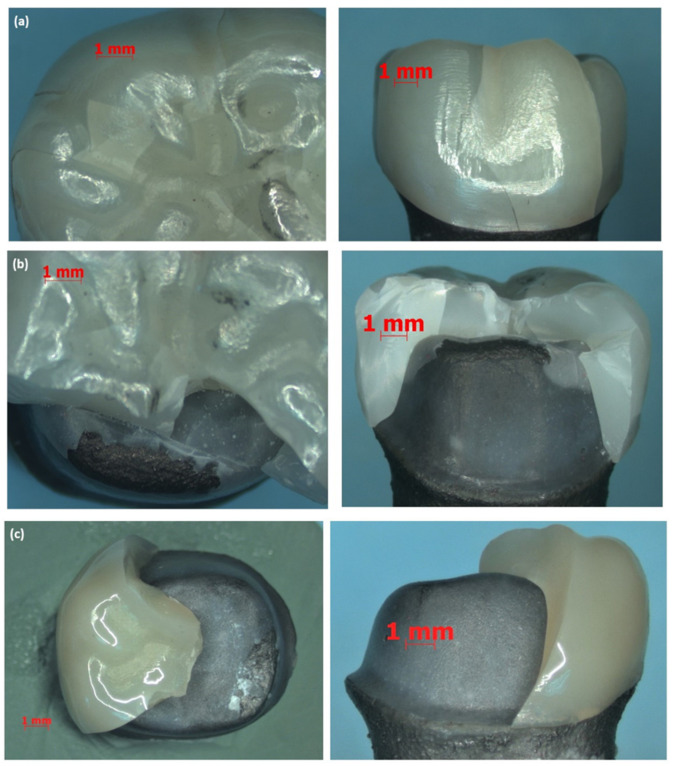

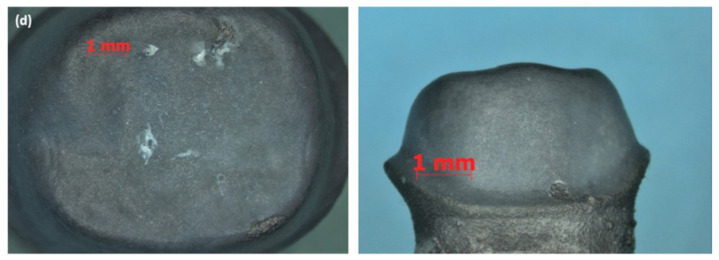
Stereomicroscopic analysis of different type of crown failures— occlusal and buccal views of the fracture types: (**a**) Type I, (**b**) Type II, (**c**) Type III, (**d**) Type IV.

Mechanical aging of the crown specimens did not induce failure in any of the crowns [37,48,51], in terms of cracks or other types of fractures. All crown specimens failed under load-to-fracture testing. The group mean (SD) and median (IQR) load-to-fracture values of Zi and ZLS crowns are presented in Table 4. The box plot (Figure 8) illustrates the spread of fracture load data for the different material—aging groups using the aforementioned statistics. The statistical analysis of the fracture load values showed significant differences between the Zi and ZLS groups, with or without aging (*p* < 0.05) (Table 4). To further analyze the differences in fracture load between and within the material groups, multiple comparisons were performed using Bonferroni post-hoc analysis. Both Zi-WMA and Zi-MA groups significantly differed from ZLS-WMA and ZLS-MA groups (*p* < 0.05); however, there were no differences found between the aged and non-aged groups within the same crown material, for both Zi and ZLS (*p* > 0.05). Table 5 shows the distribution of different types of failures among the Zi and ZLS crowns. Figure 9a–d illustrates the different types of crown failures, as seen under the stereomicroscope.

## 4. Discussion

In terms of marginal and internal gap comparison for the two materials, Zi and ZLS, part of the first null hypothesis was affirmed. However, given the significant differences in AMD values between the two material groups, this aspect of the first null hypothesis was rejected. As regards the fracture resistance of the two materials, there were significant differences between Zi and ZLS crowns, in terms of fracture resistance, with or without mechanical aging, supporting the rejection of this null hypothesis. However, since there were no differences between aged and non-aged groups of both crown materials in terms of fracture resistance, the second null hypothesis failed to be rejected.

The marginal gap found in this study for monolithic Zi crowns (37.7 ± 11.7 µm) was comparable with the mean ± SD values reported in other recent studies assessing similar restorations, as follows: 44.5 ± 7.9 µm [55], 53 ± 2 µm [60], mean range 15–47 µm [61], 26.8 ± 10.5 µm [38], 38 ± 12 µm [62], and 53 ± 7 µm [63]. The current mean internal gaps (142 ± 21 µm) seemed to be congruent with some recent studies, at 160 ± 23 µm [64] and 110–162 µm [65]. However, the values were also 30–40 µm higher than those reported in other studies [37,66]. Several factors could be responsible for these deviations, including the type of Zi material used for fabrication, luting space setting and cementation protocol, laboratory variations in the finishing of crowns, number of examined sites, and evaluation method used for the measurement of IG. Very few studies examined the AMD of Zi crowns and found values of 82–103 µm [37] and 85–133 µm [60], much less than the current values. The potential reasons for this effect could be the minimum to no adjustment of crown margins in the current study prior to evaluation of fit accuracy, the effect of sintering on the final marginal contour, and the variation in technique used for AMD assessment in studies. The results of this investigation show that careful adjustment of the crown margins after machining and sintering is imperative for minimization of positive overhangs on restorations and thus the AMDs consequently.

As for the ZLS crowns, the mean AMD values (128.1 ± 49.1µm) found in this study closely matched the results (148 ± 11 µm and 132.1 ± 39.4 µm) obtained in recent papers [31,67]. However, the current AMD values were also markedly lower than the discrepancies (235.5 ± 35.7 µm) reported in another paper [28]. The reasons for the differences could possibly be attributed to the type of abutment used for evaluation (natural tooth abutment replica in the present study versus implant abutment in the other study), crown fabrication technique employed, and software used. The mean marginal gaps in the present study (39.5 ± 7.4 µm) showed similarities with the numbers reported in a recent study, of 38.4 ± 4 µm [67], but they were also in disagreement with some others, e.g., 77.9 ± 36.7 µm [68] and 85 ± 40 µm [30]. The disparities in the mean marginal gap between the present and earlier papers and the relative small marginal gaps seen in this report might be ascribed to the CAD/ CAM system/5-axis milling machine used, milling bur condition, crystallization parameters set, laboratory technician skill and experience, fit evaluation method used, abutment preparation design/margin configuration, and the effect of no cementation.

Overall, the mean marginal gaps in this study for both Zi and ZLS crowns were well below the clinically acceptable level of 120 µm proposed by McLean and Fraunhofer, for favorable prognosis of restorations. The vertical marginal gaps were also in the range reported for ceramic crowns (3–174 µm) generally, and below the threshold of 80 µm reported for CAD/CAM ceramic crowns in recent systematic reviews [20,21]. The mean AMD values were noticeably higher than the marginal gap values in this study for both crown systems. The differences were consistent with the findings of several other studies that also investigated these two aspects of marginal fit [28,31,60,67]. The variability in the AMD values between Zi and ZLS could be related to the inherent differences in the milling process—dry milling for Zi versus wet milling for ZLS, and the use of specific types of grinding burs for each material type. The magnitude of differences between the AMD and marginal gaps could be related to the adjustments performed on the intaglio surface of the crowns as required, prior to fit evaluation to ensure good seating after examination under the microscope, but with minimal to no adjustments made on the crown-tooth marginal junction to adjust over contour to avoid risk of chipping or fracture of crown margins. Hence, the AMD values (which include the horizontal component of the marginal fit) were much higher than the vertical marginal gap values. Additionally, other factors such as the cement spacer settings, dental technician influence, CAD/CAM system used, milling machine specifications and burs, sintering and firing protocols, glazing technique, and marginal fit evaluation method may have all had an effect on the final outcome.

The increased gap in the occlusal part of the crown compared to the axial and marginal gap was consistent with the findings in many other studies [28,37,65] that also found similar differences in gap widths between the aforementioned locations generally, and this could be possibly attributed to geometry and diameter of the milling burs used in the occluso-axial line angles of the intaglio surface of the crown, determining the smallest grindable radius [69]. Another possible reason for the differences between the occlusal and axial/ marginal gaps could be due to a phenomenon that causes distortion when capturing the edges of three-dimensional structures (occluso-axial angles of prepared teeth).

A cast metal die was used as a master model in this study for evaluation of marginal and internal fit, based on several other papers that had used similar types of dies for fit evaluation of ceramic crowns [27,34,36,38,55,68,70]. An anatomical tooth preparation design complete with finish line curvatures and tooth reduction following the occlusal anatomy was used in this study, instead of the routine single-plane margins with flat occlusal surfaces used in many other recent investigations [25,34,55,70], to simulate the clinical situation better. Recent studies have shown that marginal fit of zirconia crowns is on par or even better than that of ceramo-metal crowns [55,70], which were long considered as a gold standard in fixed prosthodontics; hence, monolithic Zi crowns were used as a ‘control’ in this study to compare against the new ZLS crowns.

In the current report, an upgraded version of micro-CT, the nano-CT, was used in the evaluation of crown fit accuracy. Fit measurements were carried out at a total of 64 sites for each crown specimen, for both marginal (16 sites) and internal gaps (48 sites), based on earlier studies [28,55,63]. The pre-set sagittal and coronal slice positions on each abutment–crown sample allowed for easy identification, orientation, and performance of measurements on similar sections on each crown, thus ensuring uniformity of the process between individual samples. Three-dimensional analysis of crown-abutment complex with nano-CT facilitated the evaluation of the crown fit on the abutment tooth at any given marginal and internal location in a non-destructive manner and was also the recommended fit evaluation method for indirect fixed restorations in recent systematic review papers [20,21]. Microscopic techniques (including SEM and stereomicroscopes), on the other hand, provided for only two-dimensional assessments of marginal and/or internal gaps at the abutment–crown marginal/ internal interfaces on the cut sections with restrictions on the number of slices and measurement locations for each specimen. Additionally, destructive methods (sectioning of samples) were required for internal fit measurements, which were overcome with the nano-CT.

In this study, the fit measurement prior to cementation eliminated the influence of luting cement as a variable; however, this could be considered as a minor limitation, as the test condition did not replicate the clinical situation completely. The milled ZLS crowns were crystallized and glazed in this investigation. Some recent reports have demonstrated notable differences between the marginal gaps of pre-crystallized versus post-crystallized lithium disilicate (LDS) CAD crowns [36,71]. The potential differences in crystallization, if any, on the ZLS crowns could not be assessed in the current study, as the measurements were not performed at different stages.

In this paper, the fracture loads for both Zi and ZLS crowns (with or without cyclic loading) were higher than the values reported in most available studies and also exceeded the maximum recorded bite forces (800–1000 N) in the literature [72,73,74]. However, from a clinical standpoint, the findings need to be interpreted with caution, as the fracture loads do not completely represent failures in intra-oral use. The fracture resistance values (4677 ± 1742 N) found in this report for ZLS crowns were higher than the mean values of 3056 N to 3712 N reported in other recent papers [49,58,75] on ZLS crowns. However, the results of this study also matched the findings of some papers that found fracture strength values of 4570 ± 1242 N [76] and 4100 N [58], albeit with milled monolithic LDS crowns. It is worth mentioning that Arslan and Tosun [76] tested the fracture load of crowns on Co-Cr dies, similarly to the current paper. Few other authors [37,77,78] also reported ZLS crown fracture strength in the 2200–2500 N range with 1 mm occlusal thickness of crowns. The reasons for the disparities in the fracture strength results between current and previous ZLS studies could be due to various factors, including the cementation of crowns on metal dies, use of self-adhesive resin cementation, monolithic versus veneered crowns, selected abutment tooth type/preparation design, fabrication method/milling machine type differences, indenter (antagonist) type and diameter, examined crown thickness, and the crystallization parameters used. The general explanation for the high fracture strength of ZLS could be attributed to the presence of zirconia (ZiO_2_) in the glass matrix aiding in the transformation of the metastable tetragonal phase into a stable monoclinic phase, hence preventing the formation and propagation of the crack. The crown failure pattern noted in this study for ZLS crowns corroborated with the findings of another recent paper [58] that also found similar results. Nearly all the failures recorded in this study (13/16) were in the type IV category (severe fracture of the crown), closely matching the earlier published data where 100% (10/10) mode of failure was type IV.

As regards the fracture loads of monolithic Zi crowns, the outcomes of this study concur with the findings of previous investigations [54,55,79] that reported static fracture load values upwards of 10,000 N (10 kN) for monolithic Zi crowns with 1.5 mm occlusal thickness. Two studies [54,79] also mentioned that the full contoured Zi crowns did not break with the highest force applied (10–10.5 kN), but the actual force needed to fracture the crowns was not assessed in the studies. In this paper, a mean fracture load of 13,207 ± 4104 N was found for monolithic Zi crowns with occlusal thickness of 1.5–2 mm, in line with the projections of the aforementioned studies. With lesser occlusal thicknesses, the fracture loads in this report might have also been lower, as suggested by many other past studies [56,57,79] reporting fracture resistance values of 5700–8000 N with occlusal thicknesses of 0.5 mm to 1.2 mm. Additionally, the elastic modulus of the supporting die structure has been shown in studies to influence the fracture resistance of ceramic crowns [54,80,81]. The fracture loads recorded for posterior ceramic crowns and zirconia cores cemented on metal dies (having a higher elastic modulus) were significantly higher compared to the values noted with dentin (natural teeth) and epoxy resin dies having a lower elastic modulus [80,81]. The rationale behind this effect is, during function, forces are transmitted through the intaglio surface of the ceramic crowns to the underlying luting cement film and supporting core structure, causing stresses and deformation at these levels. The higher the elastic modulus, the greater is the stiffness and rigidity of a material and lesser the deformation of the material under a given load. Hence, rigid metal dies resist deformation under loads much better than the epoxy resin and dentin (natural teeth) and improve the fracture resistance of the brittle ceramic crowns [82]. Apart from the occlusal thickness and elastic modulus of die material, other factors such as material type, abutment preparation geometry, finish line configuration, testing conditions (no thermocycling), luting cement type, and restoration fit accuracy may have all contributed to the outcomes achieved with Zi crowns in this investigation. The failure modes of monolithic Zi crowns were spread across all types, with half of them occurring as type IV. In most cases, large fragments of the crowns were separated from the abutment tooth and the fractures occurred in the mesio-distal direction, in congruence with the pattern reported in scientific literature [41,57]. Based on the stereomicroscopic findings, the origin of failure for almost all the crowns assessed in this study, barring the ones that were completely displaced or lost (with small or no remaining parts adhering to the metal die), seemed to be at the cervical finish line, concurring with the findings of other related studies [57,79,83]. Scanning electron microscopic examination was not performed on the samples in this study for detailed fractographic analysis and is a minor limitation of the paper.

In the present report, both Zi and ZLS crowns showed no significant differences between the mechanically aged and non-aged crown specimens, in terms of load-to-failure values, similarly to the results reported in other recent investigations [37,56]. However, the results also differed with a past study [84], although it needs to be mentioned that Empress 2 and ProCAD glass ceramics were examined in that study. The reasons for the differences between the current results and the earlier outcomes could be related to the ceramic material used, the number of thermocycling and mechanical loading cycles, and cement type and crown thickness, among other factors. All the crowns (Zi and ZLS) survived the cyclic fatigue loading in this study, showing only small traces of wear on the occlusal loading surface, with no evident crack lines or fractures. These results corresponded with the observations made in other reports [37,41,48,51]. A self-adhesive resin cement was used in the current paper for cementation of crowns on the master dies. Notable differences in fracture loads of ZLS crowns have been reported by previous studies [29,49] when used with different types of cements, and the results might have also been different in this study, too, if other luting cements (glass ionomer or resin-modified glass ionomer) were used instead of or in comparison to self-adhesive resin. A single CAD/CAM system and 5-axis milling machine were used in this study for fabricating both types of crowns, thus providing standardization and eliminating the influence of software and milling unit differences on the final outcomes. A recent systematic review [85] found significant differences between the marginal and internal accuracy of CAD/CAM inlay/onlay restorations milled by 5-axis compared to 4-axis or other earlier versions, with the 5-axis performing better.

There are some deficiencies in this study that deserve discussion. Firstly, although the crown specimens were subjected to artificial aging (dynamic loading) prior to the load-to-fracture tests, they were not thermocycled in this study. A UTM set up was used for conducting the cyclic fatigue loading and static load-to-fracture tests under dry conditions, thus precluding the thermocycling of crowns. The use of a chewing simulator or other suitable device in lieu of or in addition to the UTM would have allowed for the entire thermocycling-mechanical loading (TCML) procedure to be carried out, simulating the clinical situation more closely. Additionally, the crowns post-cementation were placed in water storage for a week before the cyclic loading tests. The clinical environment could have been better mimicked with a longer water storage time and by also replicating the effect of LTD (3 h of autoclave treatment at 134 °C and 2 bar, approximately equaling 10 years of service in-vivo at 37 °C) for the Zi crowns [56,83]. Secondly, the crowns were cemented on Co-Cr metal dies in this study for fracture strength testing; use of epoxy resin or composite resin dies would have provided a more accurate reflection of the fracture resistance values in in-vivo conditions. Previous studies have highlighted some drawbacks of using natural abutment teeth for in-vitro investigations, as the teeth may fracture under high loads during fracture testing, in addition to the difficulty of standardization and comparability between the different natural teeth specimens. Although different types of metal dies have been used as supporting structures for fatigue load aging and fracture strength testing of ceramic crowns in past in-vitro studies [45,46,47,56,75,76], the set-up undoubtedly increases the failure load value of the ceramic restoration [54,81,83].

The AMD values obtained in this study were somewhat a reflection of both marginal gap and overhang combined. Most of the crown samples in both material groups demonstrated positive overhangs, whereas negative overhangs, if any, were observed at very few measurement locations in this investigation. However, these differences were not evident in the AMD values, as all values were positive, regardless of an overextended or underextended crown margin. The lack of delineation between the two types of horizontal discrepancies could be seen as a minor limitation of this paper. From a clinical point of view though, horizontal discrepancies either in the form of over-contour or step are both considered unacceptable, and the notable differences between the marginal gap and AMD values detected in this study, for both Zi and ZLS crowns, clearly identify this aspect.

Currently, CAD/CAM techniques have found widespread application for all types of prostheses—fixed, partial, and completely removable, with different types of ceramic and polymer-based materials [10,15,16,86]. Future studies comparing the fit accuracy and fracture resistance of the latest monolithic translucent Zi (partially and fully stabilized zirconia), fully crystallized ZLS, and nano-ceramic materials with the monolithic Y-TZP, LDS, and ZLS pre-crystallized glass ceramic materials are required to test the efficacy of the new materials for different types of indirect fixed restorations. It would also be useful to investigate and contrast the recently introduced heat-pressed monolithic ZLS with the milled ZLS, using the parameters examined in this study. Lastly, in-vivo studies with robust methodological designs assessing the clinical outcomes of posterior ZLS crowns in relation to the traditional materials will assist in confirming the findings of the current study.

## 5. Conclusions

Within the limitations of this study, it can be concluded that:

1. There were no significant differences between the Zi and ZLS crowns in terms of marginal and internal gaps (*p* > 0.05); however, significant differences were found in the absolute marginal discrepancies between the two materials (*p* < 0.05).
2. Mean marginal and internal gaps of both Zi and ZLS were within the ranges reported in recent systematic reviews [20,21].
3. There were significant differences in fracture loads between the Zi and ZLS crowns, regardless of mechanical aging (*p* < 0.05).

In summary, the ZLS crowns exhibited clinically acceptable marginal and internal gaps. The fracture load values of ZLS crowns, despite being lower than the monolithic Zi crowns, complied with the acceptable limit for use in clinical practice.

## Figures and Tables

**Table 1:** Nano-CT parameters.

Type	Value
VOXEL	11.111 Nm
VOLTAGE	120 kV
CURRENT	150 µA
POWER	18 W
FILTER (Cu + Al)	1 mm
GRABAR TIME	750 ms
TIME	1.45 h
ROTATION	360°
NO. OF IMAGES	2000

**Table 2:** Mean ± SD of marginal gap, internal gap and AMD of Zi and ZLS crowns (n = 16).

Material	Marginal Gap (µm)	Internal Gap (µm)	AMD (µm)
Zi	37.71 ± 11.73	141.61 ± 20.92	224.92 ± 7.33
ZLS	39.49 ± 7.42	144.85 ± 21.07	128.13 ± 49.09

**Table 3:** Independent samples’ Student’s *t*-test comparing Zi and ZLS crowns for marginal gap, internal gap, and AMD (n = 16).

Parameter		F	Sig.	T	Df	Sig. (2-Tailed)	Mean Difference	Std. Error Mean	95% Confidence Interval of the Difference	
									Upper	Lower
MG	Equal variances assumed	1.679	0.205	0.511	30	0.613	1.773	3.47	−5.314	8.859
	Equal variances not assumed	-	-	0.511	25.342	0.614	1.773	3.47	−5.369	8.915
IG	Equal variances assumed	0.084	0.774	0.436	30	0.666	3.234	7.424	−11.928	18.396
	Equal variances not assumed	-	-	0.436	30	0.666	3.234	7.424	−11.928	18.396
AMD	Equal variances assumed	0.168	0.684	−6.278	30	0.000	−96.794	15.417	−128.279	−65.309
	Equal variances not assumed	-	-	−6.278	28	0.000	−96.794	15.417	−128.375	−65.214

**Table 4:** Mean (SD), median (IQR) of the fracture load of Zi and ZLS crowns, with (MA) and without mechanical aging (WMA), in Newtons (N) (n = 8).

Material	Mean (SD)	Minimum	Maximum	Median	IQR
Zi-WMA	14,023 (2167)	10,141	16,741	13,916.50 ^a^	3361
Zi-MA	12,390 (5465)	2525	16,084	14,766 ^a^	8887
ZLS-WMA	4600 (618)	3651	5473	4489.50 ^b^	1073
ZLS-MA	4754 (2471)	1700	8400	3800 ^b^	4542

^a,b^ Different letters indicate significant statistical difference between groups.

**Table 5:** Zi and ZLS loaded crown failure types.

Material	Type I	Type II	Type III	Type IV
Zi	1	3	4	8
ZLS	0	0	3	13
